# Dengue Hemorrhagic Fever, Uttaradit, Thailand

**DOI:** 10.3201/eid0910.020681

**Published:** 2003-10

**Authors:** Jayanton Patumanond, Chamaiporn Tawichasri, Seree Nopparat

**Affiliations:** * Chiang Mai University, Chiang Mai, Thailand; † Tha Pla Hospital, Uttaradit, Thailand

**Keywords:** Dengue Hemorrhagic Fever, Dengue, Viral Infection

**To the Editor**: Dengue hemorrhagic fever (DHF) has been recognized as a disease of young children in the past. Three decades ago most reported case-patients in Thailand were 3–6 years of age (*1*). Increasing evidence shows that the age group most affected is changing (*2*). We report evidence that in Uttaradit, Thailand, the predominant age of those who acquire DHF has increased by at least 2 years during the 1990s.

Uttaradit is a province in the northern part of Thailand. DHF is endemic in Uttaradit, as it is in most parts of the country. Between 1992 and 2001, three major outbreaks of DHF occurred, in 1993, 1998, and 2001.

The number of DHF cases reported to the Provincial Health Office from January 1992 to December 2001 (classified by age groups) was used as the estimated annual DHF incidence. Case definition and categorization followed the International Statistical Classification of Diseases and Related Health Problems (ICD-10). DHF categories reported in this study included both DHF without shock and the dengue shock syndrome (the number of cases and deaths combined). Dengue fever, a milder disease manifestation, was not included.

The age distribution of DHF cases showed that, in the 1993 epidemic, children 5–9 years of age had the largest proportion of cases, whereas in 2001, the peak age of those infected was 10–14 years. The transitional stage (mean age 11.3 years) was observed in 1998.

During the observed period, the annual mean age of DHF case-patients ranged from 8.4 to 15.1 years. Despite some fluctuation, the mean age of DHF case-patients was <10 years of age before 1996. From 1997 onward, the mean age was consistently >10 years.

The incidence of DHF in children <4 years of age decreased from 586.0/100,000 in the 1993 epidemic to 197.5/100,000 in 2001. The incidence in children 5–9 years of age also decreased from 1,330.3/100,000 to 676.6/100,000 in the corresponding years. While the incidence in children 10–14 years of age remained unchanged, the incidence in those 15–24 years of age increased from 122.8/100,000 to 323.5/100,000, and from 20.0 to 52.6 per 100,000, a more than twofold increase.

Our results clearly showed that the mean age of DHF cases increased from 10.0 years in the 1993 epidemic to 11.3 years in 1998 and to 13.2 years in 2001, as a consequence of a decrease in the incidence among children <9 years, and an increase in the incidence among the older age groups. This finding was similar to what had been observed earlier in Singapore and Indonesia (*2*,*3*).

Some researchers have found that when the average number of annual dengue infections declines, the chance of persons acquiring dengue infections declines, resulting in delays in the age when a person has experienced the first, then second, dengue infection (*4*). However, in Uttaradit, as well as in other parts of Thailand, dengue infection is endemic, with large outbreaks occurring at 2- to 3-year intervals: later epidemics have also shown an increase in the overall incidence rates. Thus, this explanation is unlikely to be the reason for a shift in the age distribution of DHF in Uttaradit.

We reviewed information that indicated that the shift in age predominance could be caused by the changes in places of transmission. Among these was the study in Singapore, which proposed that an effective mosquito-control program in households had resulted in changes in which age group had the largest number of DHF cases (*5*). A significant (p<0.001) rise in seroconversion in children >6 years of age coincided with the start of formal schooling. The likelihood of dengue infection increased with time spent away from home, suggesting that the location where dengue was acquired may have changed (*5*). The recent study in Thailand also suggested that, although dengue infection may be transmitted in the home environment, transmission within schools may also be important (*6*).

The changing of the population age structure also explained the age shifting phenomenon in some studies (*7*). In Uttaradit, however, changes in the age structure of the population were small from 1992 to 2001.

The intervening effect of vaccination against Japanese encephalitis virus, a different but related flavivirus, could also explain why the mean age for most cases of DHF increased. Cross-reaction between dengue virus and Japanese encephalitis virus is well established (*8*). Vaccination against Japanese encephalitis virus may temporarily protect persons, primarily young children, against dengue infection or at least reduce its severity, resulting in a decline in the observed incidence. The cohort of these vaccine recipients were then exposed to dengue infection later in life and exhibited diseases when they shifted into an older age group. An increase of Japanese encephalitis vaccine coverage from 96% in 1995 to 100% in 2001 (*9*) appeared to confirm the above explanation. Nevertheless, areas where Japanese encephalitis vaccination had not been implemented also experienced a change in the age group with the most DHF. A final alternative explanation is the effect of herd immunity. Some researchers have observed that in places where dengue does not occur yearly, older age groups have higher rates of infection (*10*). However, dengue cases had been reported every year in Uttaradit, and the intervals between each epidemic were not long. We therefore, believed that the herd immunity hypothesis did not explain the observed changing age predominance in our study.

The mean age of DHF case-patients in Uttaradit, Thailand, increased by >3 years between 1992 and 2001. This phenomenon may be important from a public health standpoint, as community and health-related personnel may still perceive DHF as a disease of only small children and unintentionally leave older children less protected or ignored. Further study is needed to confirm that the age group shifting of DHF predominance can be explained by the changes in locations where disease transmission takes place and possibly by effective household mosquito- elimination programs.
